# Using a Novel Transfer Learning Method for Designing Thin Film Solar Cells with Enhanced Quantum Efficiencies

**DOI:** 10.1038/s41598-019-41316-9

**Published:** 2019-03-22

**Authors:** Mine Kaya, Shima Hajimirza

**Affiliations:** Department of Mechanical Engineering, Texas A&M University, 3123 TAMU, College Station, TX 77843-3123 USA

## Abstract

In this study a new method for design optimization is proposed that is based on “transfer learning”. The proposed framework improves the accuracy and efficiency of surrogate-based optimization. A surrogate model is an approximation to a costly black-box function that can be used for more efficient search of optimal points. When design specifications change, the objective function changes too. Therefore, there is a need for a new surrogate model. However, the concept of transfer learning can be applied to refit the new surrogate more efficiently. In other words insights from previous experiences can be applied to learning and optimizing the new function. We use the proposed method in a particular problem pertaining to the design of “thin film multilayer solar cells”, where the goal is to maximize the external quantum efficiency of photoelectric conversion. The results show that the accuracy of the surrogate model is improved by 2–3 times using the transfer learning approach, using only half as many training data points as the original model. In addition, by transferring the design knowledge from one particular set of materials to another similar set of materials in the thin film structure, the surrogate-based optimization is improved, and is it obtained with far less computational time.

## Introduction

Machine learning has empowered important technological developments in the last decades benefiting many engineering applications. Machine learning algorithms resemble human learning by collecting data for the task in hand and establishing reasonable connections between inputs and outputs. However, the conventional methods of machine learning start learning from scratch for every new task, unlike the way human brain normally functions. The ability of human brain to transfer knowledge among tasks can lend itself to smarter machine learning algorithms. This is officically known as transfer learning which has proven to be a promising concept in data science.

Transfer learning has received attention of data scientists as a methodology for taking advantage of available training data/models from related tasks and applying them to the problem in hand^1^. The technique has been useful in many engineering applications where learning tasks can take a variety of forms including classification, regression and statistical inference. Example of classification tasks that has benefited from transfer learning include image^2,3^, web document^4,5^, brain-computer interface^6,7^, music^8^ and emotion^9^ classification. Regression transfer has received less attention compared to transfer classification^10^. Nonetheless, there are several studies on transfer learning in regression problems such as configurable software performance prediction^11^, shape model matching in medical applications^12^ and visual tracking^13^.

Artificial neural networks (ANN) are one of the regression methods with significantly generalizable learning capabilities^14–16^. The advance of computation and parallel processing in training large ANNs have led to the very popular domain of deep learning. The multilayer structure of neural networks provides a suitable framework for knowledge transfer in both regression and classification tasks. Specifically, some of the neurons/layers (*e.g*., the hidden layers) of the structure can be shared among tasks while the remaining neurons/layers (*e.g*., the output layer) determine task specific behaviors. The former layer is generally called the *general* layer which represents similarities between different tasks and the latter is the *specific* layer^17^. This flexibility has resulted in many successful implementations of transfer learning in (deep) neural networks for applications such as wind speed prediction^18,19^, remote sensing^20^, text classification^21^ and image classification^22^.

Despite the above-mentioned applications, transfer learning in optimization problems has not been evaluated thoroughly except a few fields. There are reports of the use of transfer learning in automatic hyper-parameter tuning problems^23–26^ to increase training speed and improve prediction accuracy. Transfer learning is also suitable for the iterative nature of the engineering design where surrogate-based optimization is utilized due to the complexity of the objective function. Li *et al*.^27^ proposed a transfer learning based design space exploration method for microprocessor design. Min *et al*.^28^ investigated the use of transfer learning in aircraft design problems and demonstrated the effectiveness of the proposed algorithms. Gupta *et al*.^1^ reviewed the recent progress in transfer learning in optimization problems and categorized them as sequential, multitasking and multiform transfer optimizations. Neural networks are ideal tools for surrogate model building in complex tasks particularly for knowledge transfer, due to excellent prediction performance and the ability to handle high dimensional and highly nonlinear data^14^.

Another area where transfer learning can be useful is the optimization of different but similar black-box functions with high computational cost. This is often the case in many physical or industrial design optimization problems. Suppose one would like to determine the optimal parameters of a time-consuming function. Because the evaluation of the function is intense, the practical approach to optimization is to use past funtcion samples to approximate the function behavior and generate smarter search points. Most heuristic particle based search algorithms (e.g. Genetic algorithm, particle swarm optimization, etc.) and the well-known Bayesian optimization implicitly use a similar surface learning concept. The approximate model is called a *surrogate model*, and this approach to optimization is called *surrogate-based optimization*. The surrogate model is essentially a regression/machine learning tool. Now suppose that one is dealing with optimization of the same physical objective function but in multiple different settings. The settings can be different boundary or initial conditions, different sets of physical characteristics, different environments or materials, or any other practical variation. The functions can naturally be assumed to have similar surface patterns with unique features. As a few concrete examples, imagine designing an airfoil with optimal aerodynamic properties under different settings of speed, altitude, allowable material types, etc. Every design problem is unique, but the objective functions are correlated as they pertain to the same underlying physical function (e.g. sheer stress, drag, etc.). These correlations can be captured and used across various settings. Therefore, once a surrogate model is fit and learned for one objective function, it is expected that the knowledge can be transfered to more accurately or more efficiently fit a surrogate model for another similar function. If the process improves the efficiancy and accuracy of surrogate model fitting, then it is expected that the black-box optimization is improved in general. In other words: more optimal values can be found with less computation time.

An area where the transfer learning surrogate-based optimization can be advantageous is material design problems. High fidelity simulations are computationally costly and there are many material choices for the individual parts, resulting in different settings. In particular, we are interested in a material design problem at nano-scale related to multilayer thin film solar cells. For a fixed set of materials, the objective is to choose the dimensions of the layers. These dimensions affect the photo-electric properties of the solar cell in complex ways that are hard to analytically express or anticipate^29,30^. Therefore intense computational FDTD simulations must be used for function evalaution, and hence the design is a time consuming global optimization^31–33^. Recently, we have shown that surrogate-based optimization methods can be used to solve optimization problems of this sort, and we have established their efficacy in several thin film design problems^34–36^. Consequently, the computational costs for completing optimizations were significantly reduced compared to traditional search methods. In the present study, we aim to further demonstrate that by using transfer learning both accuracy and speed of optimizations can be additionally improved.

In this study, a novel neural-network transfer learning based optimization framework is proposed. We demonstrate that the proposed framework can expedite and improve the design of multi-layered thin film structure. We assume that at least one optimization has taken place (base case). The aim is to repeat the optimization for structures with different material choices (transfer cases). The knowledge gained from the base case is transferred to the new problem by means of neural network layers. Improvement in the prediction performance due to transfer learning is studied using the out-sample mean squared error metric. The work has therefore two novelties: 1) proposing a neural network transfer learning based optimization framework for solving complex optimization problems and, 2) using the proposed method to design a multi-layer thin film solar cell structure. The organization of this paper is as follows: first the neural network based transfer optimization method is described. Then, the thin film solar cell design is explained in section 3. The training and optimization results are presented and discussed for base case and transfer cases in section 4.

## Description of the Method

The present method enables knowledge transfer between two optimization problems using transfer learning. The method consists of a base surrogate model to be used in surrogate-based optimization, and a transfer learning framework to share the gained knowledge. Surrogates (metamodels) are regression tools to map the input space ${\mathscr{X}}$ to the output space ${\mathscr{Y}}$ using low fidelity models. The response of the surrogate model can be expressed as:

1 $$F({\bf{x}})={{\bf{y}}}_{{\bf{t}}}+\varepsilon ,$$

where ${{\bf{y}}}_{{\bf{t}}}\in {\mathscr{Y}}$ is the real output, *F*(***x***) is the objective function approximation at ${\bf{x}}\in {\mathscr{X}}$ and *ε* is the error between the real and the predicted outputs. *F* is obtained by an iterative training procedure where a training dataset of input-output pairs are fed to the regressor. As a result of the training, coefficients of the predefined metamodel (in this case the weight and biases for multilayer neural networks) are obtained.

**Figure 1:**
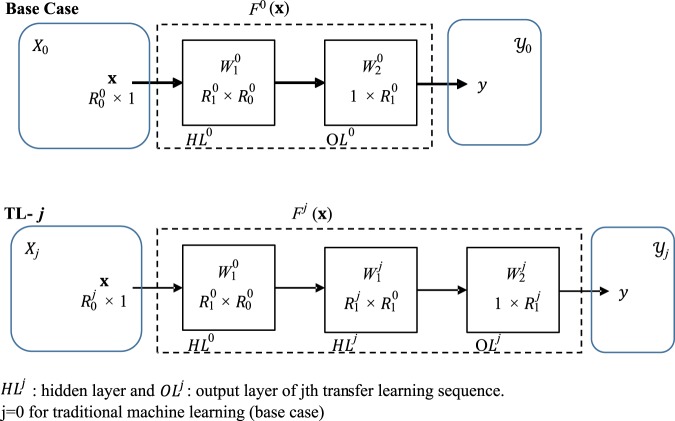
Schematic of neural network with transfer learning for a single output.

Depending on the similarity between the input-output spaces, the knowledge can be transferred from one domain (*source)* to another (*target)*. This transfer can be achieved in many ways depending on the used metamodel. Knowledge transfer using Gaussian Processes for instance can be achieved by learning a joint probability distribution and defining a common response surface^1^. Knowledge transfer in neural networks was previously recommended via shared layers^18^. In this study, we assume that we already have at least one previously trained surrogate model from a previous optimization problem. The knowledge is then transferred from this model to a new optimization problem. We propose to use a transfer learning assisted surrogate based optimization via multilayer neural networks. The aim of the present method is to improve the accuracy of the surrogate model with the same or fewer number of function evaluations. To do so, one hidden layer of the previously trained network can be borrowed as an intermediate layer. The dimensions of the new hidden layer then becomes ${R}_{1}^{0}\times {R}_{1}^{1}$ where superscripts 0 and 1 refer to the base case and the first transfer learning sequence. Therefore the input space is transformed to another space through the previously gained knowledge. This method is shown in Fig. 1. The dimensions of the input and output spaces can be same or different. In the case of different dimensions, knowledge is transferred between the matching features and the rest is treated as usual. Thus the method reduces to a dimensionality reduction approach and the accuracy of the new predictions is expected to be improved due to the similarity between the subspaces in the two different input spaces.

The output of a two layer feed forward neural network is calculated from:

2 $$y={F}^{0}({\bf{x}})={f}_{2}^{0}({W}_{2}^{0}{f}_{1}^{0}({W}_{1}^{0}{\bf{x}})),$$

where ${W}_{1}^{0}$ and ${W}_{2}^{0}$ are the coefficient matrices of the base case NN found from iterative training. ${f}_{j}(\,\cdot \,)$ is the functional operation at the *j*^th^ layer, such as sigmoid and linear function^14^. The knowledge transfer is then accomplished by transferring the hidden layer of the base case to the new case, expressed as:

3 $$y={F}^{1}({\bf{x}})={f}_{2}^{1}({W}_{2}^{1}{f}_{1}^{1}({W}_{1}^{1}{f}_{1}^{0}({W}_{1}^{0}{\bf{x}}))),$$

where ${W}_{1}^{0}$ is transferred from the base case. Training of the new case is done to find ${W}_{1}^{1}$ and ${W}_{2}^{1}$. When another case is to be optimized in the same manner, the same procedure can be repeated or the trained layer of the new case can be transferred. One drawback of the proposed method is the increase in the number of coefficients of the neural network if ${R}_{1}^{0} > {R}_{0}^{1}$ the new number of coefficients to train increases from ${R}_{1}^{1}({R}_{0}^{1}+1)$ to ${R}_{1}^{1}({R}_{1}^{0}+1)$, which may result in overfitting^37^.

The surrogate based optimization procedure starts with the design of experiment (sampling)^38^. Then, the outputs of the forward problem are evaluated at the sampled points using the simulation tool. The input/output pairs constitute the training set which is then fed to the NN trainer. All of the data is not used at once for training. Rather, it is split into training and validation. The validation error is monitored to estimate the out-sample performance of the model. The neural network is trained using one of the most widely used training algorithms, called the Levenberg-Marquardt (LM) method with Gauss-Newton approximation for Hessian^14^. For optimization, simulated annealing^39^ is used to optimize the surrogate objective function. The details of these methods can be found in the Supporting Information.

The performance of a predictive model can be quantified considering the validation set. The most common performance metric is the mean squared error defined as:

4 $$MSE=\frac{1}{{N}_{j}}\sum _{i=1}^{{N}_{j}}{\varepsilon }_{i}^{2},$$

where N is the number of data, *j* = *T*, *V* for training and validation sets respectively. *ε*_*i*_ is the error between real and approximate output for *i*^th^ instance (see equation (1)).

## Multi-Layer Thin Film Solar Cell Optimization

**Figure 2:**
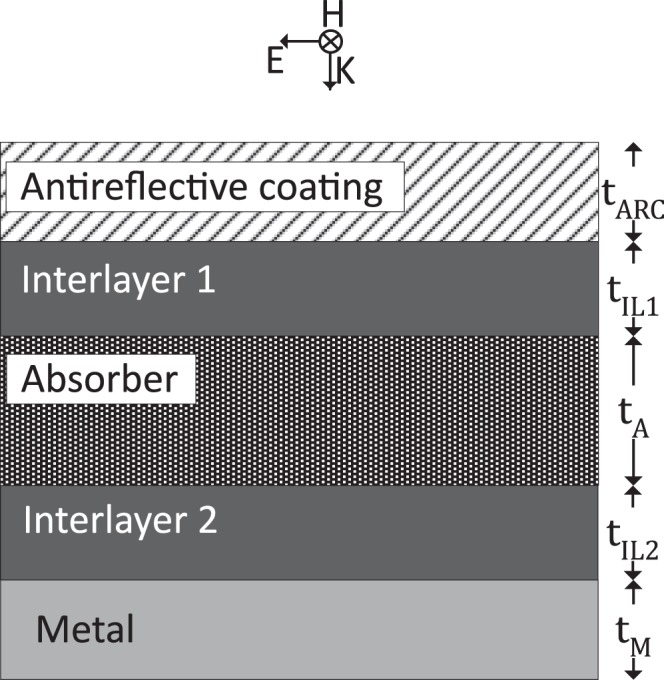
Multilayer solar cell.

We can use the explained methodology in a design optimization for multi-layer thin film solar cells. For this purpose, a simple multilayer solar cell structure is used consisting of an absorber, an antireflective coating, a back-reflector metal layer and interlayers stacked together as shown in Fig. 2. A solar cell functions on the photovoltaic principle where an electron-hole pair inside a semiconductor is created due to photon absorption. Completing the electrical circuit, the electron generates photocurrent. The interaction between the incoming light and the multilayer structure of solar cell is explained by the Maxwell’s electromagnetic theory since the characteristic length of thin film solar cells and the operation wavelengths are at the same order of magnitude (0.1−1 μm). Light-matter interaction in the near field region provides unique properties which strongly depend on the dimensions of the thin film structures. Therefore a careful optimization of the thin film geometry is required to maximize the solar cell efficiency.

In addition to the dimensions, the choice of materials used in the solar cell layers greatly affects the optical and electrical properties. On the other hand, when the material choices are included as a design variable, the optimization problem becomes a mixed-integer programming which is known to be computationally costly. Furthermore, for the present problem where the optimizations are done one by one, the optimization study should be repeated (*m*_1_×*m*_2_× … ×*m*_*d*_) times for all possible material combinations where *d* is the input space dimension and *m*_*j*_ (1 ≤ *j* ≤ *d*) is the number of choices for the *j*^th^ input. In this case, knowledge transfer between different material combination tasks is worthwhile, as similar geometries with different material combinations can have similar opto-electrical responses. In general, the initial assumption is that source and target domains are similar^27^. Although, sometimes the false similarity assumption can case negative transfer and hurt the learning^40^. Therefore the similarity assumption must be monitored and evaluated carefully.

**Figure 3:**
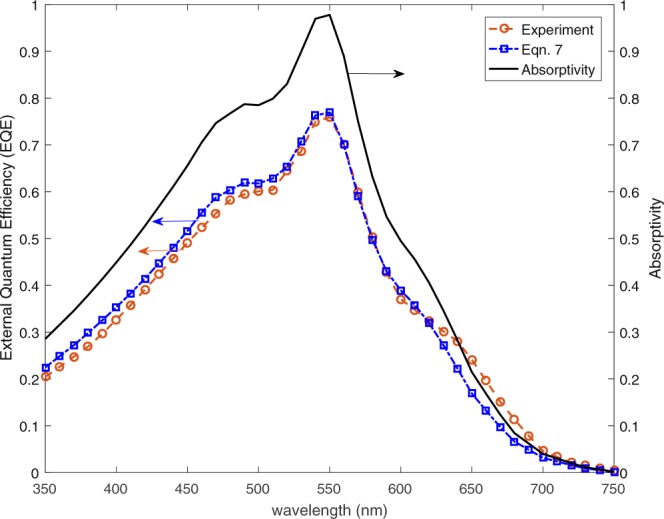
Comparison of measured and calculated EQE and simulated absorptivity profile for Ag/ZnO:Al/a-Si/ITO solar cell.

An efficient solar cell must provide desirable optical and electrical properties which can be quantified by the external quantum efficiency (EQE). EQE is defined as the ratio of number of generated electrons to the number of incident photons on the solar cell. Previously, a probabilistic expression for EQE was developed as follows^36^:

5 $${\eta }_{e}=\frac{{N}_{p}}{{N}_{i}}\frac{2{L}_{D}}{{t}_{A}}(1-\exp (-{t}_{A}/2{L}_{D}))\,$$

where *N*_*p*_ and *N*_*i*_are the number of photons absorbed and incident respectively, *t*_*A*_ is the absorber layer thickness and *L*_*D*_ is the diffusion length of the semiconductor material used as the absorber (*L*_*D*_ ≈ 100 nm). The proposed EQE model (equation (5)) was validated with experiment results^29^. The EQE of a Ag/ZnO:Al/a-Si/ITO solar cell was measured and absorbed power in the active layer was calculated using FDTD method. The same absorption profile is used to calculate EQE using equation (5) for *t*_*A*_ = 100 nm and *L*_*D*_ = 100 nm. The comparison of experiments and present calculations based on absorptivity is given in Fig. 3. FDTD simulations are performed using a commercial software by Lumerical Inc.^41^ Note that the model matches closely with the experiments for most of the relevant spectrum. The details of this probabilistic model can be found in the previous study of the authors^36^.

After doing necessary replacements, EQE becomes:

6 $${\eta }_{e}({\bf{x}})=\frac{2{L}_{D}}{hc{N}_{i}}\frac{1-\exp (-{t}_{A}/2{L}_{D})}{{t}_{A}}\,{\int }_{{\rm{\Lambda }}}^{\,}\lambda \alpha ({\bf{x}},\lambda )I(\lambda )d\lambda $$

where *λ* is wavelength, Λ is the relevant spectrum, *α*(**x**, *λ*) is absorptivity, *I*(*λ*) is AM1.5 standard spectrum and ${\bf{x}}={[{t}_{ARC},{t}_{IL1},{t}_{A},{t}_{IL2},{t}_{M}]}^{T}$ is the geometry vector.

The optimization problem then becomes:

$$\mathop{{\rm{\max }}}\limits_{{\bf{x}}}\,{\eta }_{e}({\bf{x}}),$$

7 $${{\bf{x}}}_{{\bf{L}}} < {\bf{x}} < {{\bf{x}}}_{{\bf{U}}}.$$

The surrogate objective function can be calculated using the surrogate model of absorptivity, *f*(**x**, *λ*) ≈ *α*(**x**, *λ*) instead of EQE, since EQE can be calculated using the spectral absorptivity (see equation (6)). Therefore, the computational cost is further reduced by considering the wavelength as an input variable of the surrogate model and calculating EQE accordingly.

Once an optimization study was carried out for a base case, the present method can be used to optimize a solar cell structure with the same geometry but different materials. For example, once we optimize an ITO/ZnO/P3HT:PCBM/MoO_3_/Al solar cell structure as a base case, less effort should be necessary for the optimization of a five layer solar cell consisting of different materials. For this purpose, a base case and transfer cases are selected as follows:

8 $$\begin{array}{rcl}{{\bf{x}}}_{{\boldsymbol{S}}} & = & {[{t}_{ITO},{t}_{ZnO},{t}_{P3HT:PCBM},{t}_{Mo{O}_{3}},{t}_{Al}]}^{T}\\ {{\bf{x}}}_{{\boldsymbol{TL}}-{\bf{1}}} & = & {[{t}_{ITO},{t}_{Si{O}_{2}},{t}_{aSi},{t}_{A{l}_{2}{O}_{3}},{t}_{Al}]}^{T}\\ {{\bf{x}}}_{{\boldsymbol{TL}}-{\bf{2}}} & = & {[{t}_{S{i}_{3}{N}_{4}},{t}_{PEDOT:PSS},{t}_{PCDTBT:PCBM},{t}_{A{l}_{2}{O}_{3}},{t}_{Al}]}^{T}\end{array}$$

These materials are widely used in thin film solar cells. TL-1 case was previously designed and optimized by the authors^36,42^ using surrogate based and direct optimization methods. TL-2 is optimized for the first time.

As shown in Fig. 1, the base case is optimized by using traditional surrogate-based optimization methods. Then the hidden layer of the trained model is transferred to other cases. In the next section, the results are presented for the base case and transfer cases with emphasis on the training and validation performances (mean squared error). We will also discuss computational cost as the required number of simulation iterations.

## Results and Discussion

### Base Case

**Figure 4:**
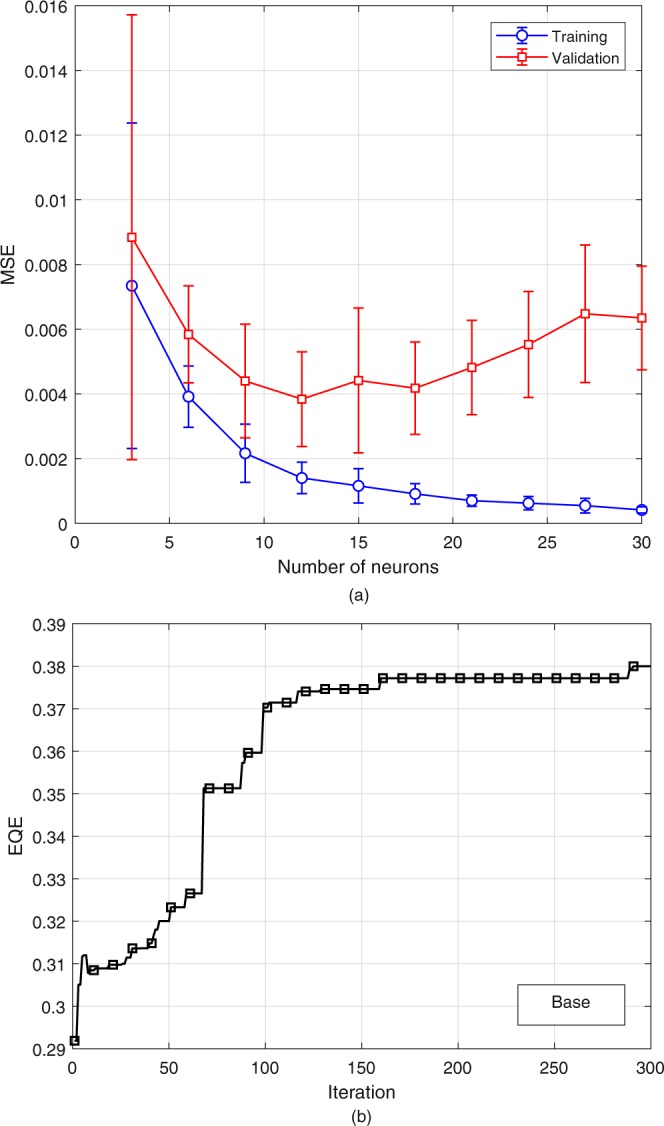
**(a**) Variation of mean squared error for training and validation data sets with respect to the number of neurons in the hidden layer of NN for base case, (**b**) Evolution of EQE during the optimization for the base case.

The training of the base case is done using 1000 data points with 750 of them used as the training set and the rest for validation. The number of neurons in the hidden layer is determined based on the principle of minimum validation error as follows: The in-sample and out-sample errors are recorded as the number of neurons in the hidden layer is increased and the network configuration providing the minimum out-sample error is selected to be used in the optimization. This procedure is repeated 10 times to eliminate the possibility of training algorithm being trapped in local optima. Optimization is also repeated 10 times using all NN models obtained. This results in 10 possible optimal points. These points are run throught the high-fidelity (FDTD) model, and the highest function value is selected accordingly. The number of neurons in the hidden layer for the base case is selected as 12 based on the results in Fig. 4a. Then the optimization is done using the NN models with 12 neurons using all the generated models. Full-fidelity optimization is also done using the software in order to validate the results (See Table 1). The optimized values are in a good agreement with a maximum 5% error. The evolution of EQE during surrogate-based optimization iterations is presented in Fig. 4b. Note that the best reported EQE in Table 1 is obtained using simulations so discrepancies between this value and that of Fig. 4b are expected.

**Table 1:** Optimization results for Base, TL-1 and TL-2 cases.

Case Name	*N*_*sims*_ [−]	*t*_*ARC*_ [nm]	*t*_*IL*1_ [nm]	*t*_*A*_ [nm]	*t*_*IL*2_ [nm]	*t*_*M*_ [nm]	*EQE* [−]
Base – NN-based	1,000	76	19	79	12	100	0.370
Base - Direct	6,900	77	20	80	10	95	0.371
TL-1 – NN-based – w/TL	500	31	20	65	20	102	0.371
TL-1 – NN-based – no TL	1,000	29	19	65	20	101	0.370
TL-1 – Direct	9,200	30	19	65	20	103	0.372
TL-1 – Reference^42^	4,600	30	16	62	20	50	0.361
TL-2 – NN-based – w/TL	500	40	5	98	5	95	0.355
TL-2 – NN-based – no TL	1,000	38	7	95	5	100	0.352
TL-2 – Direct	5,520	42	5	100	5	97	0.360

### Transfer Cases

**Figure 5:**
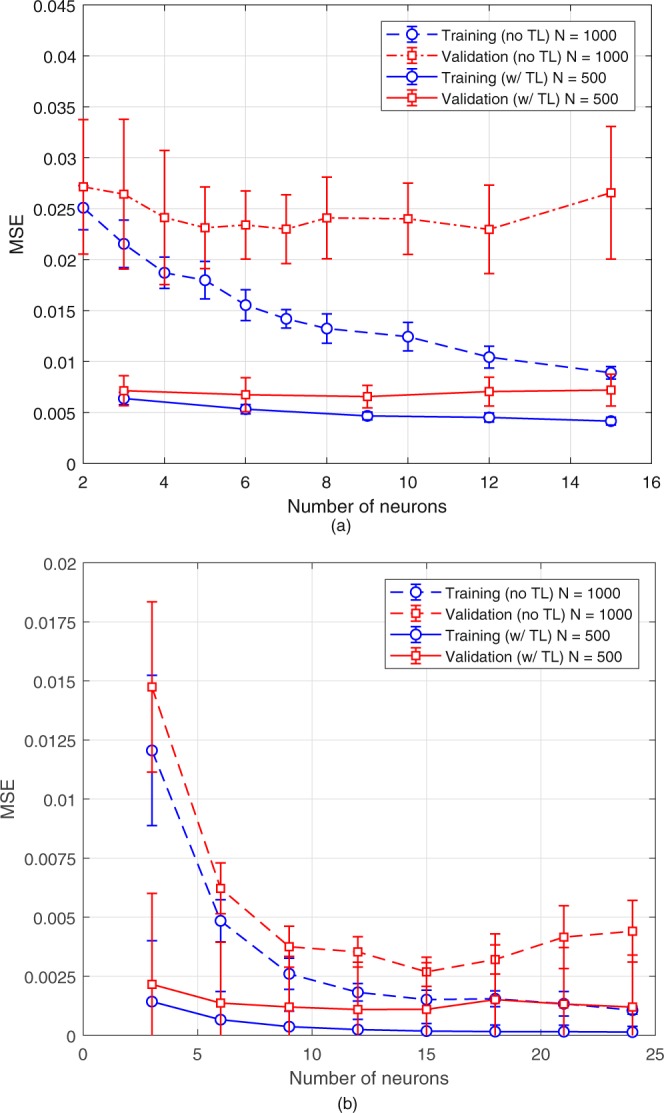
Results for (**a**) TL-1 (ITO-SiO_2_-aSi-Al_2_O_3_-Al) and (**b**) TL-2 (Si_3_N_4_-PEDOT:PSS-PCDTBT:PCBM-Al_2_O_3_-Al) without the transfer layer (no TL, dashed lines) using 1000 data points and with transfer layer (w/TL, solid lines) using 500 data points.

In order to demonstrate the proposed approach, two material sets different from the base case are considered. These sets are represented by vectors **x**_**TL**−1_ and **x**_**TL**−2_. First, the same steps as in the base case are followed without the transfer learning framework as a comparison. In these cases, 1000 data points are used where 750 of them are used as the training set and the rest is used for validation. Then training is repeated for the transfer learning cases using equation 3 with 500 new data points where 375 of them are used as the training set and the rest is used for validation. The prediction performances using transfer learning are presented and compared with the traditional method in Fig. 5a,b.

Figure 5 shows the effectiveness of the transfer learning method. The smallest out-sample MSE of no TL case in TL-1 is more than 3 times larger than the largest out-sample MSE w/TL case even though the number of data is half of the no TL case. Furthermore, although the improvement in TL-2 case is not as significant as in TL-1, using the transfer layer reduces error to almost half of the TL-2 (no TL). The reason of this less significant improvement is that the validation error of TL-2 case without transfer layer is similar to that of the base case. On the other hand, the validation error of TL-1 (no TL) case is ~5 times larger than that of the base case. As can be seen from Fig. 5b, the most significant improvement in validation error is obtained when 3 neurons is used where the largest deviation between errors of TL-2 and base cases is observed. Therefore the relation between the deviation between errors of transfer and base cases suggest that the more accurate the base case is, the more the validation error is reduced. Furthermore, if the base case is less accurate than the transfer cases, prediction performance can even become worse. This is known as negative transfer which is an undesirable phenomenon in transfer learning applications.

**Figure 6:**
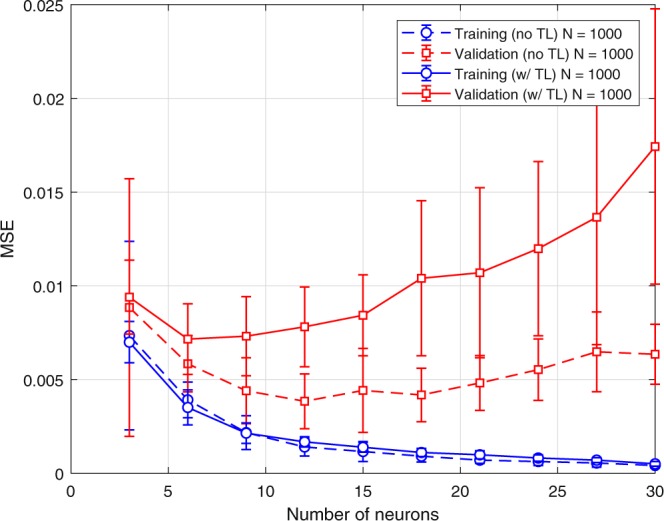
Negative Transfer: Comparison of MSE of no TL Base case (dashed) and w/TL from TL-1 (solid).

The effect of negative transfer on prediction accuracy is illustrated in Fig. 6 by switching the base and TL-1 cases where the hidden layer of TL-1 (ITO-SiO_2_-aSi-Al_2_O_3_-Al) is transferred to the base case (ITO-ZnO-P3HT:PCBM-MoO_3_-Al). As seen from Fig. 6, the training MSE does not change as expected; however, the validation error significantly increases since the transferred layer is adopted from a less accurate model.

In TL-1 case, 12 and 9 neurons are selected for no TL and w/TL respectively for optimization. The results are compared with the previous optimization studies for the same 5-layer a-Si solar cell^36,42^. Similarly, in TL-2 case, 15 and 12 neurons are selected for no TL and w/TL respectively for optimization.

**Figure 7:**
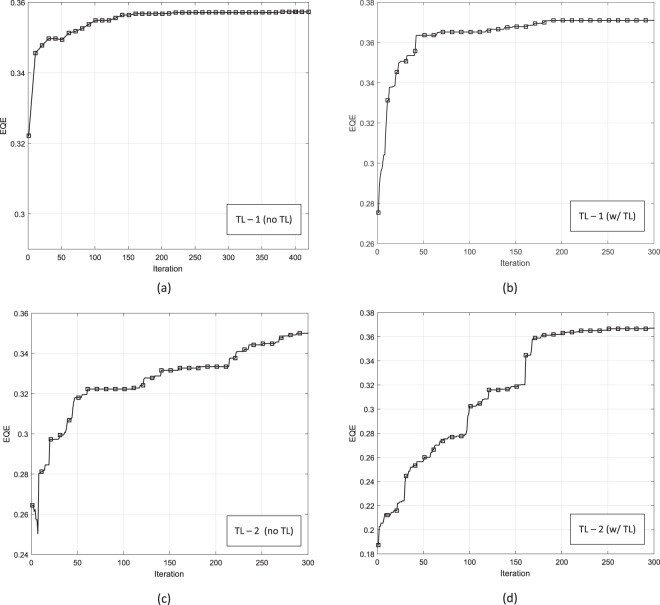
Evolution of EQE during optimization for (**a**) TL-1, w/out TL, (**b**) TL-1 w/TL, (**c**) TL-2 w/out TL (**b**) TL-2 w/TL.

The results obtained using transfer learning are in a good agreement with the direct optimization results for both cases. The optimized geometry in TL-1 case is also very close to the results from the previous study^36^. In the other study^42^, a regression-tree based optimizer is used as well as simulated annealing on direct FDTD simulations to find the optimal solution. However, since the objective function in this study^42^ is slightly different than the present objective function, a deviation between the results of these two studies is expected. The present study achieved a slightly higher EQE than that the previous result^42^. The optimization results are presented in Table 1 and evolutions of EQE are presented in Fig. 7.

Results show that equivalent EQEs can be obtained from an amorphous silicon and an organic P3HT:PCBM solar cell. EQE of PCDTBT:PCBM solar cell is lower than the others because the longer wavelengths where PCDTBT:PCBM can absorb more than a-Si and P3HT:PCBM are ignored in EQE calculation. EQE is calculated between *λ* = 300–750 nm for all cases for consistency.

## Conclusion

In this paper, a novel methodology of multilayer neural network based transfer optimization for design problems was presented. The proposed method was applied to a case study where a multilayer thin film solar cell was to be optimized for the best external quantum efficiency. The results showed that the prediction accuracy can be improved using transfer learning. Furthermore, the number of high fidelity function evaluations during surrogate based optimization can be decreased without sacrificing the accuracy.

## Data Availability

The datasets generated during the current study are available from the corresponding author on reasonable request.
